# Supplementary material for: Combined use of karyotyping and copy number variation sequencing technology in prenatal diagnosis
Source: PeerJ. 2022 Dec 5;10:e14400. doi: 10.7717/peerj.14400 (PMC9745786; doi:10.7717/peerj.14400)
Supplement: Supplemental Information 1 — #NT, nuchal translucency; NF, thickened nuchal fold; NIPT, non-invasive prenatal testing; AMA, advanced maternal age; CNV, copy number variation; TOP, termination of pregnancy; PGD, preimplantation genetic diagnosis. [file peerj-10-14400-s001.docx]

| Case No. | Detailed clinical indicator(s)^＃^ | Karyotype | CNV-seq results | Classification | Follow-up |
| --- | --- | --- | --- | --- | --- |
| 1 | Fetal congenital heart disease (endocardial cushion defect) | 47, XN, +21 | seq[GRCh38]dup(21)(q11.2q22.3)  NC_000021.9:g.12965809_46699983dup | Pathogenic | TOP |
| 2 | NIPT high-risk for trisomy 21 | 47, XN, +21 | seq[GRCh38]dup(21)(q11.2q22.3)  NC_000021.9:g.12965809_46699983dup | Pathogenic | TOP |
| 3 | NT 4.6mm; Maternal serum  screening high risk | 47, XN, +21 | seq[GRCh38]dup(21)(q11.2q22.3)  NC_000021.9:g.12965809_46699983dup | Pathogenic | TOP |
| 4 | NT3.3-3.5mm; NIPT high-risk for trisomy 21 | 47, XN, +21 | seq[GRCh38]dup(21)(q11.2q22.3)  NC_000021.9:g.12965809_46699983dup | Pathogenic | TOP |
| 5 | Fetal congenital heart disease | 46,XN,rob(21;21)(q10;q10) | seq[GRCh38]dup(21)(q11.2q22.3)  NC_000021.9:g.12965809_46699983dup | Pathogenic | TOP |
| 6 | NT 5.0mm; NIPT high-risk for trisomy 21 | 47, XN, +21 | seq[GRCh38]dup(21)(q11.2q22.3)  NC_000021.9:g.12965809_46699983dup | Pathogenic | TOP |
| 7 | Maternal serum screening high risk | 47, XN, +21 | seq[GRCh38]dup(21)(q11.2q22.3)  NC_000021.9:g.12965809_46699983dup | Pathogenic | TOP |
| 8 | NT 4.3mm | 47, XN, +21 | seq[GRCh38]dup(21)(q11.2q22.3)  NC_000021.9:g.12965809_46699983dup | Pathogenic | TOP |
| 9 | Maternal serum screening high risk; History of adverse reproductive | 47, XN, +21 | seq[GRCh38]dup(21)(q11.2q22.3)  NC_000021.9:g.12965809_46699983dup | Pathogenic | TOP |
| 10 | NT 3.8mm | 47, XN, +21 | seq[GRCh38]dup(21)(q11.2q22.3)  NC_000021.9:g.12965809_46699983dup | Pathogenic | TOP |
| 11 | NT 4.5 mm | 47, XN, +21 | seq[GRCh38]dup(21)(q11.2q22.3)  NC_000021.9:g.12965809_46699983dup | Pathogenic | TOP |
| 12 | NT 4.7mm | 47, XN, +21 | seq[GRCh38]dup(21)(q11.2q22.3)  NC_000021.9:g.12965809_46699983dup | Pathogenic | TOP |
| 13 | NIPT high-risk for trisomy 21 | 47, XN, +21 | seq[GRCh38]dup(21)(q11.2q22.3)  NC_000021.9:g.12965809_46699983dup | Pathogenic | TOP |
| 14 | NT 5.5 mm | 47, XN, +21 | seq[GRCh38]dup(21)(q11.2q22.3)  NC_000021.9:g.12965809_46699983dup | Pathogenic | TOP |
| 15 | NIPT high-risk for trisomy 21 | 47, XN, +21 | seq[GRCh38]dup(21)(q11.2q22.3)  NC_000021.9:g.12965809_46699983dup | Pathogenic | TOP |
| 16 | NT 3.1 mm | 47, XN, +21 | seq[GRCh38]dup(21)(q11.2q22.3)  NC_000021.9:g.12965809_46699983dup | Pathogenic | TOP |
| 17 | NIPT high-risk for trisomy 18 | 47, XN,+18 | seq[GRCh38]dup(18)(p11.32q23)  NC_000018.10:g.10001_80259271dup | Pathogenic | TOP |
| 18 | Maternal serum screening high risk | 47, XN,+18 | seq[GRCh38]dup(18)(p11.32q23)  NC_000018.10:g.10001_80259271dup | Pathogenic | TOP |
| 19 | Maternal serum screening high risk | 47, XN,+18 | seq[GRCh38]dup(18)(p11.32q23)  NC_000018.10:g.10001_80259271dup | Pathogenic | TOP |
| 20 | AMA; Positive for ultrasonographic soft markers | 47, XN,+18 | seq[GRCh38]dup(18)(p11.32q23)  NC_000018.10:g.10001_80259271dup | Pathogenic | TOP |
| 21 | NF 9.1mm, cerebellar hemispheric separation | 46,XN,rob(13;14)(q10;q10) | seq[GRCh38]dup(13)(q12.11q34)  NC_000013.11:g.18925860_114344403 dup | Pathogenic | TOP |
| 22 | NT 3.8mm | 45, XN,  rob(14;21)(q10;q10) | seq[GRCh38]dup(21)(q21.1q22.3)  NC_000021.9:g.19047682_46680088dup | Pathogenic | TOP |
| 23 | NT 3.4mm | 47, XYY | seq[GRCh38]dup(Y)(p11.32q12)  NC_000024.10:g.1_57217415dup | Pathogenic | Term birth, no obvious abnormal |
| 24 | NIPT high-risk for sex chromosome | 47, XXX | seq[GRCh38]dup(X)(p22.33q28)  NC_000023.11:g.10001_156030895dup | Pathogenic | TOP |
| 25 | NIPT high-risk for sex chromosome | mos 45,X[22]/46,XY[8] | seq[GRCh38]del(Y)(p11.32q12)(mos)  NC_000024.10:g.1_57217415del  seq[GRCh38]del(Y)(q11.221q11.23)  NC_000024.10:g.16428120_26653853del | Pathogenic | TOP |
| 26 | NIPT high-risk for sex chromosome | mos 45,X[33]/46,XX[17] | seq[GRCh38]del(X)(p22.33q28)  NC_000023.11:g.10001_156030895del | Pathogenic | TOP |
| 27 | NT 3.0mm | 45, X | seq[GRCh38]del(X)(p22.33q28)  NC_000023.11:g.10001_156030895del | Pathogenic | TOP |
| 28 | NIPT high-risk for sex chromosome | mos45,X[24]/47,XXX[16] | seq[GRCh38]del(X)(p22.33q28)(mos)  NC_000023.11:g.10001_156030895del | Pathogenic | TOP |
| 29 | Maternal serum screening high risk | 47,XY,+?mar | seq[GRCh38]dup(Y)(p11.32q12)  NC_000024.10:g.1_57217415dup | Pathogenic | TOP |
| 30 | Fetal ultrasound structural abnormalities | 47,XXY | seq[GRCh38]dup(X)(p22.33q28)  NC_000023.11:g.10001_156030895dup | Pathogenic | TOP |
| 31 | Maternal serum screening high risk; NIPT high-risk for sex chromosome | 47,XXY | seq[GRCh38]dup(X)(p22.33q28)  NC_000023.11:g.10001_156030895dup | Pathogenic | TOP |
| 32 | Fetal congenital heart disease; Paternal chromosome abnormalities | 46,XX,der(4) t(4;16)(q35;q21q24) | seq[GRCh38]dup(16)(q21q24.3)  NC_000016.10:g.62946097_90093592 dup | Pathogenic | TOP |
|  |  |  | seq[GRCh38]del(4)(q35.1q35.2)  NC_000004.12:g.182998848_189518846del |  |  |
| 33 | NIPT high-risk for chromosome 8 | 46,XN,der(12)t(8;12)(q22;q24.1) | seq[GRCh38]dup(8)(q22.1q24.3)  NC_000008.11:g.96809900_145070385 dup  seq[GRCh38]del(12)(q24.33)  NC_000012.12:g.132174657_133200976del | Pathogenic | TOP |
| 34 | NIPT high-risk for chromosome 9 | 46,XN,del(9)(p22) | seq[GRCh38]del(9)(p24.3p22.1)  NC_000009.12:g.208454_18950991del | Pathogenic | TOP |
| 35 | AMA | 46,XN,der(9)del(9)(p23p24.3)dup(9)(p13.1p23) | seq[GRCh38]del(9)(p24.3p23)  NC_000009.12:g.200000_13580001del  seq[GRCh38]dup(9)(p23p13.1)  NC_000009.12:g.13580001_38780003 dup | Pathogenic | TOP |
| 36 | NIPT high-risk for 18 chromosome | 45,XN,-18[9]/46,XN,?r(18)(p11.32q21.31)[48] | seq[GRCh38]del(18)(q21.31q23)  NC_000018.10:g.58192768_80259271del  seq[GRCh38]del(18)(p11.32)  NC_000018.10:g.140000_1159999del | Pathogenic | TOP |
| 37 | PGD | 46,XN,t(12;14)(q23;q32) | seq[GRCh38]del(22)(q11.21q11.21)  NC_000022.11:g.18892487_20332477del | Pathogenic | TOP |
