# Supplementary material for: Combined use of karyotyping and copy number variation sequencing technology in prenatal diagnosis
Source: PeerJ. 2022 Dec 5;10:e14400. doi: 10.7717/peerj.14400 (PMC9745786; doi:10.7717/peerj.14400)
Supplement: Supplemental Information 2 — #CNV, copy number variation; TOP, termination of pregnancy; AMA, advanced maternal age; NIPT, non-invasive prenatal testing; NT, nuchal translucency; FGR, fetal growth restriction. [file peerj-10-14400-s002.docx]

| Case No. | Detailed clinical indicator(s)^＃^: | CNV-seq results | Classification | Follow-up |
| --- | --- | --- | --- | --- |
| 38 | Fetal congenital heart disease | seq[GRCh38]del(22)(q11.21)  NC_000022.11:g.18892487_21465711del | Pathogenic | TOP |
| 39 | Maternal serum screening high risk | seq[GRCh38]del(5)(p15.33p15.1)  NC_000005.10:g.20001_17939891del | Pathogenic | TOP |
|  |  | seq[GRCh38]dup(7)(q34q36.3)  NC_000007.14:g.141680201_159335973dup | Pathogenic |  |
| 40 | Chromosomal abnormalities of pregnant woman | seq[GRCh38]del(1)(p36.33p36.32)  NC_000001.11:g.884621_2823435del | Pathogenic | TOP |
| 41 | Maternal serum screening high risk | seq[GRCh38]del(8)(p23.3p23.1)  NC_000008.11:g.210001_7082478del | Pathogenic | TOP |
| 42 | Maternal serum screening high risk | seq[GRCh38]del(17)(p12)  NC_000017.11:g.14196684_15516686del | Pathogenic | Term birth,  no obvious abnormality |
| 43 | Fetal lateral ventricle enlargement; AMA | seq[GRCh38]del(16)(p13.3)  NC_000016.10:g.29555975_30178708del | Pathogenic | TOP |
| 44 | Maternal serum screening high risk | seq[GRCh38]del(16)(p11.2)  NC_000016.10:g.28799003_29077303del | Pathogenic | TOP |
| 45 | Bowel echo enhancement, fetal congenital heart disease | seq[GRCh38]del(2)(p16.3)  NC_000002.12:g.50880234_51125144del | Pathogenic，maternally inherited | TOP |
| 46 | AMA; Single umbilical artery, left kidney absent | seq[GRCh38]del(15)(q11.2)  NC_000015.10:g.22595660_23102647del | Pathogenic | TOP |
| 47 | AMA; NIPT high-risk for other chromosome | seq[GRCh38]dup(15)(q11.2q13.1)  NC_000015.10:g.23374854_28294854dup | Pathogenic | TOP |
| 48 | Maternal chromosome abnormalities | seq[GRCh38]del(1)(p36.33p36.32)  NC_000001.11:g.884621_2823435del | Pathogenic | After birth，  Obvious abnormality |
| 49 | AMA; Chromosomal microdeletion syndrome of previous fetus | seq[GRCh38]del(9)(p24.3p24.1)  NC_000009.12:g.200000_6760000del  seq[GRCh38]dup(20)(p13p12.3)  NC_000020.11:g.79360_8139353dup | Pathogenic | TOP |
| 50 | Fetal lateral ventricle enlargement | seq[GRCh38]del(16)(p13.3)  NC_000016.10:g.35880_147065del | Pathogenic，maternally inherited | Term birth,  no obvious abnormality |
| 51 | Maternal serum screening high risk | seq[GRCh38]dup(22)(q11.21)  NC_000022.11:g.18892488_21125711dup | Pathogenic，maternally inherited | Term birth,  no obvious abnormality |
| 52 | Induction of labor due to congenital heart disease of previous fetus | seq[GRCh38]del(22)(q11.21) NC_000022.11:g.20362586_214461821del | Pathogenic，maternally inherited | Term birth,  no obvious abnormality |
| 53 | AMA | seq[GRCh38]del(X)(p22.33p22.32)  NC_000023.11:g.2781959_5541959del | Pathogenic，maternally inherited | Term birth,  no obvious abnormality |
| 54 | NIPT high-risk for other chromosome | seq[GRCh38]del(15)(q13.2q13.3)  NC_000015.10:g.30767797_32147799del | Pathogenic | TOP |
| 55 | NT 2.8mm; Maternal serum screening high risk | seq[GRCh38]del(16)(p13.11p12.3)  NC_000016.10:g.15426143_18086143del | Pathogenic，maternally inherited | Term birth,  no obvious abnormality |
| 56 | NIPT high-risk for sex chromosome | seq[GRCh38]del(X)(p22.31)  NC_000023.11:g.6541959_8171959del | Pathogenic，maternally inherited | Term birth,  no obvious abnormality |
| 57 | NIPT high-risk for sex chromosome | seq[GRCh38]del(X)(p22.31)  NC_000023.11:g.6541959_8171959del | Pathogenic，maternally inherited | Term birth,  no obvious abnormality |
| 58 | NIPT high-risk for sex chromosome | seq[GRCh38]del(X)(p22.31)  NC_000023.11:g.6537110_8167062del | Pathogenic | Term birth,  no obvious abnormality |
| 59 | Maternal serum screening high risk | seq[GRCh38]dup(16)(p13.11)  NC_000016.10:g.15026143_16646143dup | Likely pathogenic | Term birth,  no obvious abnormality |
| 60 | NT 5.0mm | seq[GRCh38]del(X)(p22.31p22.2)  NC_000023.11:g.8422532_10917281del | Likely pathogenic | Cleft lip, pleural effusion, TOP |
| 61 | FGR | seq[GRCh38]dup(X)(q25q26.1)  NC_000023.11:g.129446225_130254419dup | Likely pathogenic | Term birth,  no obvious abnormality |
| 62 | Absence of nasal bone; Pregnancy with gestational diabetes | seq[GRCh38]del(13)(q13.2q13.3)  NC_000013.11:g.34643644_35422587del | Likely pathogenic | TOP |
| 63 | Short long bone | seq[GRCh38]dup(2)(q37.3)  NC_000002.12:g.236551357_242077849dup | Likely pathogenic | Term birth,  no obvious abnormality |
| 64 | Nasal bone dysplasia; Maternal serum screening high risk | seq[GRCh38]del(20)(p13)  NC_000020.11:g.79360_1119357del | Likely pathogenic | Term birth,  no obvious abnormality |
| 65 | Nasal bone dysplasia; Maternal serum screening high risk | seq[GRCh38]dup(16)(p13.11)  NC_000016.10:g.14946143_16206143dup | Likely pathogenic | Term birth,  no obvious abnormality |
| 66 | History of bearing child with chromosome abnormalities | seq[GRCh38]del(16)(p12.2)  NC_000016.10:g.21928680_22428679del | Likely pathogenic | Term birth,  no obvious abnormality |
