# Supplementary material for: Combined use of karyotyping and copy number variation sequencing technology in prenatal diagnosis
Source: PeerJ. 2022 Dec 5;10:e14400. doi: 10.7717/peerj.14400 (PMC9745786; doi:10.7717/peerj.14400)
Supplement: Supplemental Information 3 — #CNV-seq, copy number variation sequencing; VUS, variants of unknown significance; NIPT, non-invasive prenatal testing; AMA, advanced maternal age; FGR, fetal growth restriction; TOP, termination of pregnancy. [file peerj-10-14400-s003.docx]

| Case No. | Detailed clinical indicator(s)^＃^ | CNV-seq results | Classification | Follow-up |
| --- | --- | --- | --- | --- |
| 67 | Intra-abdominal cystic mass | seq[GRCh38]dup(10)(q23.1)  NC_000010.11:g.83520244_84440244dup | VUS | birth asphyxia |
| 68 | Fetal hydronephrosis | seq[GRCh38]dup(6)(p12.3)  NC_000006.12:g.4439766_47192264dup | VUS | Stillbirth, Ultrasound examination showed that fetal hydronephrosis increased to 22 mm |
| 69 | Maternal serum screening high risk | seq[GRCh38]dup(3)(p14.1)  NC_000003.12:g.67990858_69030849dup | VUS | Term birth,  no obvious abnormality |
| 70 | Fetal head facial skin edema | seq[GRCh38]dup(14)(q24.2)  NC_000014.9:g.71413284_73153292dup | VUS | Term birth,  no obvious abnormality |
| 71 | High-risk NIPT results for other chromosome | seq[GRCh38]dup(18)(p11.32)  NC_000018.10:g.560001_2160000dup | VUS | Term birth,  no obvious abnormality |
| 72 | Maternal serum screening high risk; History of adverse reproductive | seq[GRCh38]dup(15)(q13.2q13.3)  NC_000015.10:g.30094195_32639720dup | VUS  maternally inherited | Term birth,  no obvious abnormality |
| 73 | AMA | seq[GRCh38]dup(21)(q21.1q21.3)(mos)  NC_000021.9:g.18847682_25687688dup | VUS | Term birth,  no obvious abnormality |
| 74 | FGR of the first-born | seq[GRCh38]del(6)(q12)  NC_000006.12:g.65270108_66470107del | VUS | Term birth,  no obvious abnormality |
| 75 | Absence of nasal bone | seq[GRCh38]del(X)(q27.3) NC_000023.11:g.144236894_144498449  del | VUS | Term birth,  no obvious abnormality |
| 76 | Fetal head facial skin edema | seq[GRCh38]dup(6)(q16.3)  NC_000006.12:g.102812125_103232125  dup | VUS | Term birth,  no obvious abnormality |
| 77 | FGR | seq[GRCh38]del(7)(q35)  NC_000007.14:g.145533158_146707380  del | VUS | TOP |
| 78 | AMA; positive for ultrasonographic soft markers | seq[GRCh38]dup(2)(p12)  NC_000002.12:g.78404583_79623963dup | VUS | Term birth,  no obvious abnormality |
| 79 | Encephalocele of the previous fetus | seq[GRCh38]dup(8)(q21.3)  NC_000008.11:g.91027773_92267772dup | VUS | Term birth,  no obvious abnormality |
| 80 | Fetal persistent left superior vena cava | seq[GRCh38]dup(18)(q23)  NC_000018.10:g.76768045_77988044 dup | VUS | Term birth,  no obvious abnormality |
| 81 | AMA | seq[GRCh38]dup(13)(q14.3)  NC_000013.11:g.52605865_53745865dup | VUS | Term birth,  no obvious abnormality |
| 82 | Down syndrome of previous fetus; Absence of nasal bone | seq[GRCh38]dup(13)(q21.1)  NC_000013.11:g.54965865_56345866dup | VUS | Term birth,  no obvious abnormality |
| 83 | Right aortic arch, ventricular septal defect | seq[GRCh38]del(15)(q11.2)  NC_000015.10:g.22813068_23113068del | VUS | Term birth,  no obvious abnormality |
| 84 | Fetal ventriculomegaly | seq[GRCh38]del(7)(q31.1)  NC_000007.14:g.111159944_111459944  del | VUS | Term birth,  no obvious abnormality |
| 85 | NIPT high-risk for other chromosome | seq[GRCh38]dup(4)(q12q13.1)  NC_000004.12:g.57313834_61854282dup | VUS | Term birth,  no obvious abnormality |
| 86 | The last fetus was induced due to fetal hydrops | seq[GRCh38]dup(8)(p21.2)  NC_000008.11:g.24042487_25342484dup | VUS | Term birth,  no obvious abnormality |
| 87 | AMA | seq[GRCh38]dup(3)(q24)  NC_000003.12:g.144521158_146182213 dup | VUS | Term birth,  no obvious abnormality |
| 88 | Maternal serum screening high risk | seq[GRCh38]dup(12)(q23.2q23.3)  NC_000012.12:g.102406222_103866222 dup | VUS | Term birth,  no obvious abnormality |
| 89 | NT:3.3mm; History of adverse reproductive | seq[GRCh38]del(1)(q21.1)  NC_000001.11:g.145675059_146055003 del | VUS | Term birth,  no obvious abnormality |
| 90 | Fetal lateral ventricle enlargement | seq[GRCh38]dup(7)(q36.1q36.2)  NC_000007.14:g.152722915_153722915 dup | VUS,  maternally inherited | Term birth,  no obvious abnormality |
| 91 | Twins (one survived and one stopped developing) | seq[GRCh38]dup(X)(p22.31)  NC_000023.11:g.6521959_8171959dup | VUS | Term birth,  no obvious abnormality |
| 92 | History of adverse reproductive | seq[GRCh38]dup(X)(p22.31)  NC_000023.11:g.6581960_8131959dup | VUS | TOP |
| 93 | Pregnancy with gestational diabetes; Missed screening time | seq[GRCh38]dup(X)(p22.31)  NC_000023.11:g.6521960_8171959dup | VUS | Term birth,  no obvious abnormality |
| 94 | AMA; Maternal serum screening high risk | seq[GRCh38]dup(X)(p22.31)  NC_000023.11:g.6521960_8191959dup | VUS | Term birth,  no obvious abnormality |
| 95 | AMA | seq[GRCh38]dup(X)(p22.31)  NC_000023.11:g.6521960_8171959dup | VUS | Term birth,  no obvious abnormality |
| 96 | AMA | seq[GRCh38]dup(X)(p22.31) NC_000023.11:g.6537110_8167527dup | VUS | Term birth,  no obvious abnormality |
| 97 | FGR, widening of cerebellar medullary cistern | seq[GRCh38]dup(1)(p36.33p36.32)  NC_000001.11:g.1768561_4799940dup | VUS | Term birth,  no obvious abnormality |
| 98 | Prenatal diagnosis requirements for couples | seq[GRCh38]del(2)(q12.1q12.1)  NC_000002.12:g.102823541_104263542 del | VUS | Term birth,  no obvious abnormality |
| 99 | Consanguineous marriage | seq[GRCh38]dup(X)(q27.2) NC_000023.11:g.141245872_141671842 dup | VUS,  maternally inherited | lost follow-up |
