# Supplementary material for: Combined use of karyotyping and copy number variation sequencing technology in prenatal diagnosis
Source: PeerJ. 2022 Dec 5;10:e14400. doi: 10.7717/peerj.14400 (PMC9745786; doi:10.7717/peerj.14400)
Supplement: Supplemental Information 4 — AMA, advanced maternal age; NA, not available (absent or unrecorded) [file peerj-10-14400-s004.docx]

| Case No. | Detailed clinical indicator(s)^＃^ | Karyotype | Origin | Follow-up |
| --- | --- | --- | --- | --- |
| 100 | AMA | 46, XN,t(8;18)(p21;q11.2) | NA | Term birth, no obvious abnormality |
| 101 | Carrier of translocated chromosome:46,XY,t(10;12)(q11;p13) | 46, XN,t(10;21)(q11;p13) | Paternal | Term birth, no obvious abnormality |
| 102 | Carrier of translocated chromosome:46,XY,t(8;11)(p12;q21) | 46, XN,t(8;11)(p12;q21) | Paternal | Term birth, no obvious abnormality |
| 103 | AMA | 47,XN,+mar | NA | Term birth, no obvious abnormality |
| 104 | Carrier of translocated chromosome:46,XX,t(3;12)(p22;q24.2) | 46, XN,t(3;12)(p23;q24.2) | Maternal | Term birth, no obvious abnormality |
| 105 | AMA | 46, XN,inv(7)(p15q11.2) | NA | Term birth, no obvious abnormality |
| 106 | Maternal serum  screening high risk | 46,XN,t(3;12)(p14;p13) | NA | Term birth, no obvious abnormality |
| 107 | Carrier of translocated chromosome:46,XX,t(1;10)(q42,q26); NIPT high-risk | 46, XN,t(1;10)(q42;q26) | Maternal | Term birth, no obvious abnormality |
| 108 | AMA | 46,XN,?del(5)(p15.3) | NA | Term birth, no obvious abnormality |
| 109 | AMA | 46,XN,t(8;9)(p22;q22) | NA | Term birth, no obvious abnormality |
| 110 | Increased echogenicity in enteroids | 45,XN,rob(13;14)(q10;q10) | Maternal | Term birth, no obvious abnormality |
| 111 | Fetal chromosomal abnormalities | 46, XN,inv(5)(p13q15) | NA | Term birth, no obvious abnormality |
| 112 | AMA | 46, XN,inv(8)?(p23,1q13) | NA | Term birth, no obvious abnormality |
| 113 | AMA | 46,XN,inv(6)(p11q15) | NA | Term birth, no obvious abnormality |
| 114 | AMA | 46,XN,t(2;18)(q13;q21.3) | NA | Term birth, no obvious abnormality |
| 115 | AMA | 46,X,inv(Y)(p11.3;q12) | NA | Term birth, no obvious abnormality |
| 116 | AMA | 46,XN,?add(15) | NA | Term birth, no obvious abnormality |
| 117 | Carrier of chromosome Roche translocation | 45,XN,rob(14;21)(q10;q10) | Maternal | Term birth, no obvious abnormality |
| 118 | fetal ultrasound structural abnormality | 45,XN,-18[2]/46,XN[48] | NA | Term birth, no obvious abnormality |
| 119 | History of adverse pregnancy and childbirth | 46,XN,t(1;13)(q42;q32),t(6;10)(q25;q24)[15]/46,XN,t(6;10)(q25;q24)[50] | NA | Term birth, no obvious abnormality |
